# Ergonomic Design of Manual Assembly Workstation Using Digital Human Modeling

**DOI:** 10.5334/aogh.3256

**Published:** 2021-06-25

**Authors:** Pouya Alipour, Hadi Daneshmandi, Mohammad Fararuei, Zahra Zamanian

**Affiliations:** 1 Department of Ergonomics, School of Health, Shiraz University of Medical Sciences, Shiraz, Iran; 2 Research Assistant Professor of Health Sciences (By Research), Research Center for Health Sciences; Research Institute for Health, Shiraz University of Medical Sciences, Shiraz, Iran; 3 Department of Epidemiology, School of Health, Shiraz University of Medical Sciences, Shiraz, Iran; 4 Department of Occupational Health Engineering, School of Health, Shiraz University of Medical Sciences, Shiraz, Iran

## Abstract

**Background:**

Manual assembly workers are exposed to risk factors of musculoskeletal disorders. The most important risk factor among the workers is static and awkward posture. This study aimed to the ergonomic design of manual assembly workstation using Digital Human Modeling (DHM).

**Methods:**

This cross-sectional study was conducted among manual assembly workers. Data was gathered *via* 1) demographic/occupational questionnaire, 2) The Persian version of the Nordic General Questionnaire (P-NMQ), 3) Rapid Upper Limb Assessment (RULA) using Kinect sensor, 4) Hierarchical Task Analysis (HTA), 5) Idea Rating Sheet (IRS), 6) Anthropometric data of the participants, 7) CATIA software and RULA technique.

**Results:**

The results of the evaluations showed that in the design of most workstations of assemblers in Shiraz electronics industries, complete ergonomic principles were not observed, and the implementation of targeted ergonomic interventions in them is necessary.

**Conclusion:**

The prevalence of musculoskeletal symptoms is high among manual assembly workers. The RULA technique showed that the designed manual assembly workstation using DHM effectively could improve the subjects’ awkward postures.

## Background

Ignoring ergonomic factors in workplaces might cause many diseases and problems such as work-related musculoskeletal disorders (WMSDS) for workers [1,2,3]. However, according to estimates provided by NIOSH, musculoskeletal disorders rank the second most frequent disorder after respiratory diseases in the terms of severity and prevalence in workplaces [4]. Also, Guo et al. (2004) stated that a quarter of American workers are suffering from musculoskeletal disorders [5].

Based on ergonomic principles, the posture and movement of workers provide important information to diagnose the risk of musculoskeletal disorders in the workplace [6,7]. Moreover, the workstation is one of the most important factors affecting people’s posture while they are working. One of the main tasks of ergonomics specialists is to redesign the workstation. The ergonomic workstations design implies through different approaches such as 1) improving the quality of productivity, working life, and production 2) modification of working spaces to make services easier and more speed along with better-maintained operations 3) change in working methods including automation and task assignment between operator and machine 4) Controlling physical factors such as heat, cold, sound, vibration, and light. These approaches are being applied to increase efficiency, productivity, and safety. Besides, they would be beneficial to make applications easier, reduce human errors, stress and fatigue, improve workplace comfort of workers and eventually job satisfaction and acceptance [8,9].

Human simulation software can be used to implement ergonomic needs in the design and optimization of workstations or the entire production system. Besides, there are several emerging technologies supporting human-centered simulation based on ergonomic validation in the workplace. Such tools allow us to simulate the places and tasks even before the workplace is physically present. Ergonomic principles are applied in Digital Human Models (DHM) in the early stages of design for preventive research [10,11].

These tools provide a fast and virtual representation of the role of the human in a simulated workplace. They can be used to identify ergonomic problems and prevent the risk of musculoskeletal disorders. If we use the desired posture, the plan can be implemented in the real environment. CATIA (computer-aided three-dimensional interactive application) is one of the most widely used software which can provide a human simulation based on anthropometric data. By using CATIA, virtual mannequins in their standard positions can be put in pre-designed workstations and can predict the desired amount of power and strength based on body postures. This information can be provided by ergonomists long before the start of the job [12].

In the light of the foregoing about postural assessment in real workplaces and the importance of ergonomic workstation design for assemblers based on their anthropometric data and the expert user’s opinions, a study on this context is urgently necessary. Hence, few studies have been performed on posture evaluation using the Kinect sensor. Also, no study has been conducted using computer simulations and worker’s opinions on assemblers in Iran; therefore, the present study was conducted aiming at the ergonomic design of the workstation of assemblers in the Shiraz electronics industry using Digital Human Modeling (DHM).

## Methods

The cross-sectional study was carried out on assembly-line workers who had at least one year of work experience. The participants who were underlying diseases or affecting the musculoskeletal disorder excluded from the study. All participants attended the study voluntarily and they signed informed consent forms before taking part in the study. It should be noted that the research was performed according to the Helsinki Declaration of 1964 and revised in 2008. Besides, this study was approved by the ethics committee of Shiraz University of Medical Sciences (*IR.SUMS.REC.1398.1411*).

### Data collection tools

The data were collected using the following tools:

1. ***Demographic/occupational questionnaire***: The questionnaire contained questions about age, weight, height, job experience, daily working hours, gender, marital status, and education level.
2. ***P-Nordic Musculoskeletal Questionnaire (P-NMQ)***: The general NMQ was used to examine the reported cases of MSDs symptoms in different body regions among the study population. The validity and reliability of the Persian version of NMQ were assessed by Choobineh et al., (2004) [13]. We only assessed the musculoskeletal symptoms which had been observed during two months later. The participants were provided with the questionnaires in their workplace and were asked to complete the questionnaires during the work shift while performing their operations.
3. ***Hierarchical Task Analysis (HTA)*:** HTA describes the activity or workflow are being analyzed in terms of a hierarchy of goals, sub-goals, operations, and plans. An HTA may stand on its own or be integrated with additional task analyses. As the main benefit of an HTA, it could allow us to make further distribution of the relationships between the used cases (parent task) and subtasks through a numbering scheme.
4. ***Idea Rating Sheet (IRS)***: IRS is one of the participatory ergonomics methods. It is an effective voting mechanism used for idea selection. This method is used when we want to achieve democratic voting from the contest participants. Judges, facilitators, and other involved ones (e.g., spectators) participate in the voting process.
  After discussing the main ideas, all members of teams have been asked to fill in the idea rating sheet (one idea per sheet). All participants read every idea rating sheet and considered other team’s ideas, and record their opinions on a scale of “strong agreement,” “Agreement,” “Neutral,” “Disagreement,” “Strong Disagreement” or “Confusion.” Participants signed each sheet and meanwhile, they dotted and may choose to add brief comments on the idea sheets. When the dotting process came to end, facilitators (organizers or judges) collected sheets and sorted them by topic and agreement level.
5. ***The Rapid Upper Limb Assessment (RULA) technique***: One of the most popular observational methods is RULA. The examiner should rate a static key posture of the worker based on direct observation or a picture. This evaluation is based on an estimation of the main upper body, trunk, and neck joint angles. Each joint angle is associated with a joint score according to a predefined range of angles. These joint scores lead to final grand scores and recommendations. In this study, the body posture was assessed using Microsoft Kinect v2 sensor and K2RULA, a semi-automatic RULA evaluation software in real manual assembly workplace and online mood.
6. ***Anthropometric data***: To design the manual assembly workstation, including the table and chairs, it was necessary to collect information about the anthropometric dimensions of the assemblers as the subjects of study. In this part of the study, 16 parameters were examined based on the standard definitions of anthropometric. The measurements had been calibrated in centimeters. Then, the required database to design the assembly plant was prepared.
7. ***Computer-Aided Three-dimensional Interactive Application (CATIA)*:** To design a workstation, a computer-aided three-dimensional interactive application (CATIA) was used. CATIA is a multi-platform software suite for computer-aided design (CAD), computer-aided manufacturing (CAM), computer-aided engineering (CAE), and PLM and 3D, developed by the French company DassaultSystèmes. RULA has been used in this research to link the available functions in CATIA.

## Implementation of the study

In the first phase, the prevalence of musculoskeletal symptoms was determined by the P-NMQ. Also, the working postures of the participants were assessed using the RULA technique and a Kinect sensor.

In the second phase, the HTA was used for task analysis among participants. Then, anthropometric dimensions of the subjects were measured based on the standard definitions of anthropometric. Besides, opinions of experts and users about workstation design were collected using the IRS.

In the third phase, an ergonomic workstation was designed in CATIA. Also, working postures were analyzed in this software using the RULA technique.

Finally, to evaluate the effectiveness of the designed workstation in CATIA, the grand scores of the RULA technique derived from the first and third phases were compared.

## Statistical analysis

Data were analyzed by SPSS software version 22 using descriptive analysis, including mean, standard deviation, frequency, and percentage. The 5th, 50th, and 95th percentiles of the subject’s body dimensions were also determined. Wilcoxon statistical test was used to compare the RULA scores before derived from Kinect and after derived from CATIA design. P < 0.05 was considered to be statistically significant.

## Results

Designing an ergonomic workstation for assemblers based on anthropometric data and opinions of experts and users, as well as posture evaluation using Kinect sensor, computer simulation, and opinions of assemblers. All workers in the assembly department participated in the present study (n = 47).

***1) Demographic/occupational questionnaire***: The average age of the subjects was 34.72 years and the average height in the standing position was about 170 cm. 70% of the population in the study were men, and all of them were shift work and have been involved with musculoskeletal disorders at various times in the later year (***Table 1***).

**Table 1:** Qualitative demographic data of the subjects (n = 47).


VARIABLES		FREQUENCY	PERCENT

**Sex**	Female	14	30

	Mail	33	70

**Marital Status**	Single	16	34

	Married	31	66

**Education**	Diploma	6	69.6

	Associate Degree	8	17.4

	Bachelor’s Degree and higher	33	13

**Number of Children**	0	29	61

	1	12	26

	2 and more	16	13

**Employment Status**	Official	0	0

	Contractual	0	0

	Company	47	100

**Gender of employees in the shift system**	female	33	100

	mail	13	93

**Symptoms of Musculoskeletal Disorders**	Daily	10	21.7

	Weekly	14	30.4

	Monthly	12	39.1

	Annually	11	7.8

**Duration of Musculoskeletal Disorders**	No day	0	0

	One to seven days	30	65

	Eight to thirty days	0	0

	More than thirty days	6	13

	Everyday	11	22


***2) P-Nordic Musculoskeletal Questionnaire (P-NMQ)***: The Persian version of the Nordic questionnaire was completed by the staff working in the light assembly hall. The highest prevalence of symptoms of musculoskeletal disorders in the later 12 months was related to the neck (71%), shoulders (61.3%), and sitting and waist (54.8%), respectively. The highest prevalence of these disorders in women was in the neck (85.7) and shoulders (71.4) and for men in the neck (72.7%) and seating and low back (66.7%).

**Figure 1:**
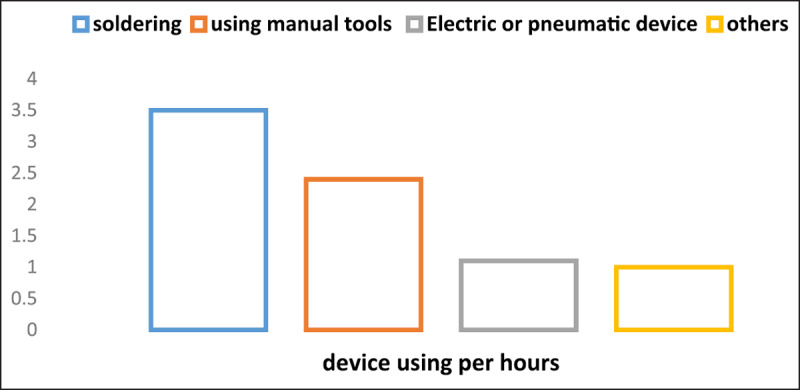
Time analysis of assembly job.

***3) Hierarchical Task Analysis (HTA)***: First, with the help of two assembly specialists with 20 years of experience, the HTA diagram of jobs was drawn on paper. The working hours using the case tool were then measured by a stopwatch. Then, with the help of digital tools (such as Microsoft Office Word and Creately.Com), the original diagram was drawn. The time analysis determines what percentage of the total working time constitutes each component of the job (***Figure 1***).

***4) Idea Rating Sheet (IRS)***: Four-hour training class about the importance and application of ergonomics, safety, and health issues was held for the staff of the assembly department. At the end of the session, the individuals were divided into groups of four to five individuals and asked to discuss the issues raised and their application in the workplace, the results are shown in ***Table 2***.

**Table 2:** Comments received from the groups in order of priority.


ROW	COMMENTS

*1*	Desk design tailored to people’s jobs.

*2*	Ergonomic chair design.

*3*	Reduce work stress and organizational pressures from managers.

*4*	Embedding space on the desk for personal items.

*5*	Provide adequate lighting.

*6*	Install high-efficiency and silent ventilation.

*7*	Proper footrest design.

*8*	Design of colored and standard tools.

*9*	Providing personal wardrobes for bags and personal items.


* Note: The sentences are summarized and standardized as much as possible.

***5) The Rapid Upper Limb Assessment (RULA) technique*:** Posture evaluation results were obtained with the help of the Kinect sensor and K2RULA software provided by the Italian Polytechnic University. Ninety percent of the studied jobs are in the priority level of corrective action three and four.

***6) Anthropometric data*:** The variables required for the ergonomic design of the assembler workstation are presented in ***Table 3***. For sitting height, sitting work surface height, and standing work height, the upper limit (95th percentile of men) and the lower limit (5th percentile of women) have been reported. The rest of the items not listed are designed to be fully customizable.

**Table 3:** Variables required for workstation Design.


DIMENSIONS	VARIABLE SIZE (cm)

Sitting depth	33.24

Sitting width	42.00

Height of forearm support	28.49

Lateral space of the foot	64.00

Foot vertical space	69.99

Front foot space	68.02


**Figure 2:**
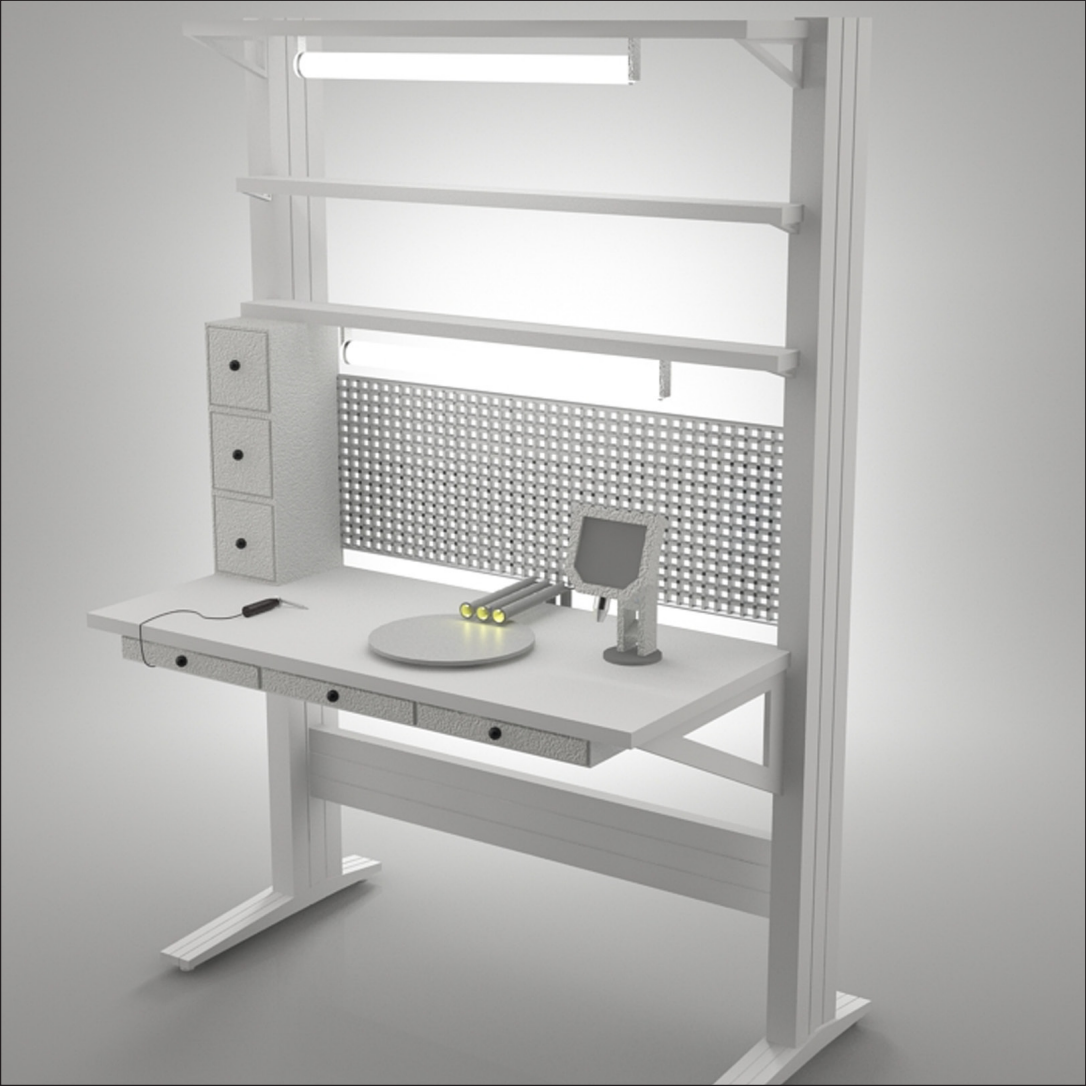
Full view of the proposed workbench for assemblers (in this image, a sample of ventilation along with lighting is also suggested and designed).

***7) Computer-Aided Three-dimensional Interactive Application (CATIA)*:** After collecting staff feedback, hierarchical and temporal analysis of tasks, determining the dimensions required for ergonomic workstation design, and a preliminary design of the workstation were prepared in DMAX 3D design software. These designs include a two-tier local lighting workstation, several drawers for personal tools and equipment, a rotating disk for grinding electrical boards during assembly and soldering, the placement of ergonomic nozzles, and a digital microscope, and the design as shown in ***Figure 2***. Local ventilation and local lighting were designed to reduce annoying shadows.

**Figure 3:**
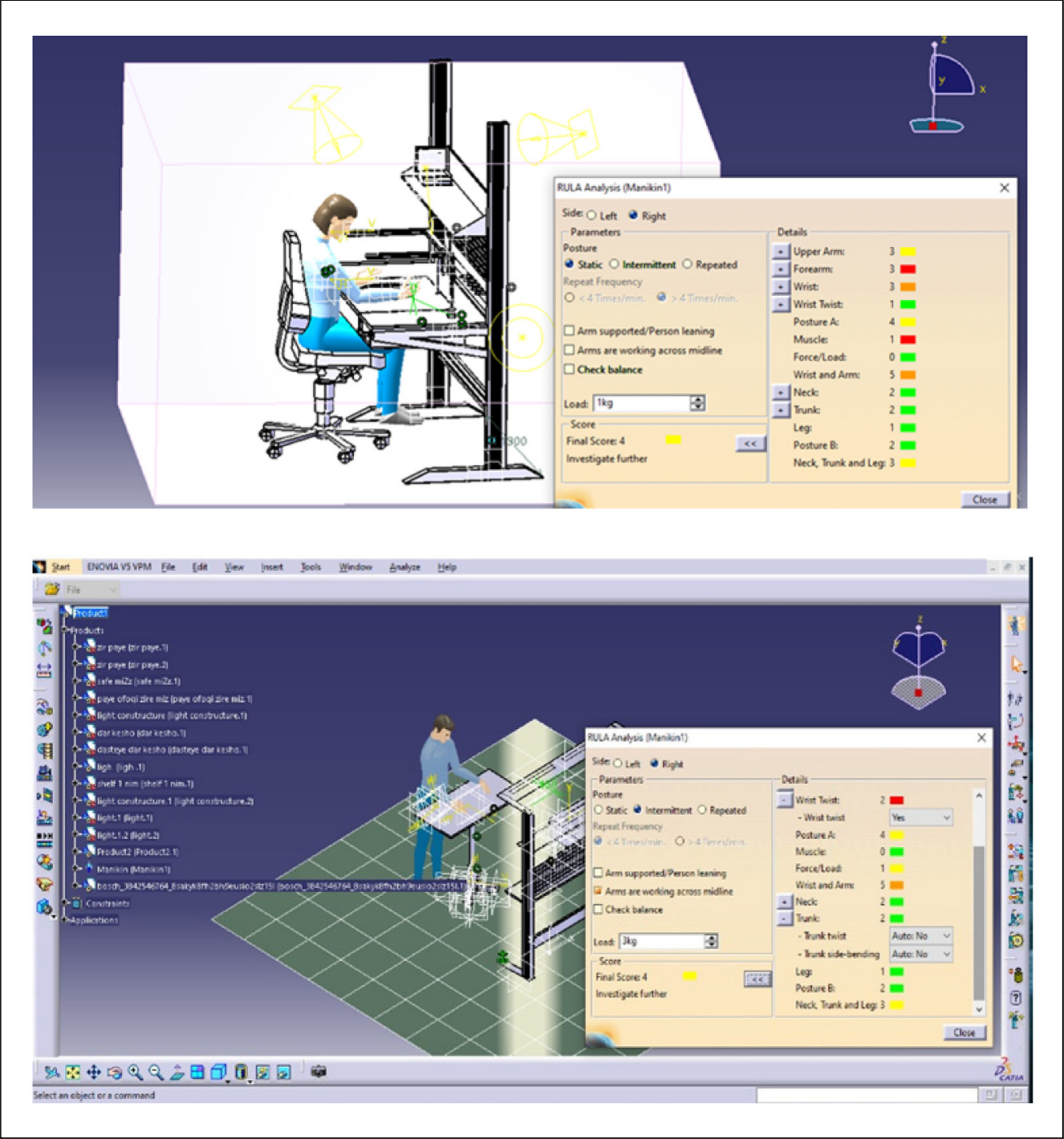
An example of how RULA is reported in CATIA.

After holding the second meeting with the employees and obtaining their opinions, the final design of the workstation was produced in CATIA software and the simulated work posture was evaluated using the RULA method. ***Figure 3*** shows an example of how to report a RULA posture evaluation.

Posture evaluation guide simulated by RULA method in CATIA software:

1 and 2: (green) determines that the posture is acceptable if not maintained or repeated for a long time.
3 and 4: (yellow) indicates that more research is needed and changes may be needed.
5 and 6: (orange) indicates that research and changes are needed soon.
7: (red color) indicates that research and changes are needed immediately.

Comparison of Kinect RULA scores and CATIA RULA scores using the Wilcoxon test shows that between the Kinect RULA scores and the CATIA RULA scores in the arm, forearm, wrist, trunk/foot area as well as there is a statistically significant correlation between the total score (p < 0.05) (***Table 4***).

**Table 4:** Comparison of RULA scores from Kinect and CATIA.


BODY AREAS	RULAfromCATIA		RULAfromKINECT	*P-VALUE
	
	MIDDLE (INTERMEDIATE RANGE)		MIDDLE (INTERMEDIATE RANGE)	

**Arms**	(5–3) 4		(1–1) 1	0.005

**Forearms**	(2–1) 1		(2–3) 2	0.015

**Wrist**	(1.75–2.75) 2		(4–3) 4	0.011

**Neck**	(3–2) 5/2		(4–1) 3	0.569

**Foot and Trunk**	(1.75–2) 2		(6–5) 5.5	0.005

**Final Score**	(3–2.75) 3		(7–5) 6	0.004


* Wilcoxon signed-rank test.

## Discussion and conclusion

The findings of this study showed that the nature of work defined for women in electrical assemblies limited in the micron dimensions. Therefore, women had to bring their heads and necks very close to the table to solder and microscopically wash the board for several hours. In addition, in some cases, they should rotate for working on several boards.

Prevalence of musculoskeletal disorders in women was 57.1% in one or both legs and they have averagely lost seven days for the musculoskeletal problems. The reason of this prevalence was mainly the distance between women›s soles and the ground. Unfortunately, women›s desks have been purchased without considering the anthropometric sizes of women (almost always the work equipment are being providing only based on the relative average size of men).

It has been found that assemblers spend most of their working time with a position in which their neck has been bent forward or backward. In the studies of Charles [14], Grieco [15], Sun Yan [16], Aghilinejad [17], Daneshmandi [18], and Maimaiti [19], it has been asserted that the musculoskeletal disorders are more prevalent in the neck and shoulders of assemblers.

The top 10 tasks of assembler were selected using Hierarchical Task Analysis (HTA) with the help of specialists working in the manual Assembly, but for security reasons, these results were not be allowed to be reported. Three tasks for women were 1) assembling the thin board, 2) working with a microscope, and 3) connecting the channels with a total score of RULA 6 and 7, which must be corrected as soon as possible. To perform these three tasks required high concentration, women have to bend their torso, neck and head. Additionally, the surface area of the assembly boards is 20 square centimeters, and focusing on these tiny boards needs to bring the forearm and the hands closer together. Being in this position for long time may lead to a squatting body shape in assemblers. Because the dimensions of the boards were unchangeable; the design for postural corrections was shifted from sitting position to standing one.

The main tasks of men were 1) assembly of the board to the chassis 2) assembly of the chassis 3) assembly with the pneumatic device, and the RULA score for all these tasks was six. This indicates that corrective action should be taken against these poor conditions as soon as possible. The multi-faceted geometric shape of the chassis and its rather high weight and the long time of the assembly process caused the muscles to work statically.

Eswaramoorthi M et al., (2010) conducted a study by HTA method. The finding suggested that the main cause of musculoskeletal disorders were the weight of tools and table height as two key parameters making ergonomic stresses [20]. In the IRS method, the content of the training classes was adjusted based on the importance of ergonomic design, risk factors, MSDs injuries, and solutions to prevent musculoskeletal disorders. The main function of the people’s opinions were similar to trainers of the classes. Bonnardel (2020) made similar observations in a study focused on the types of brainstorming to perform a creative design [21]. He suggested that to avoid these similarities, it is better to get people’s ideas before training. The items that were mostly discussed in the present study sessions were related to musculoskeletal problems, personal complaints, requests to buy a table, chairs, work problems. It was also stated that neck pain and back pain were the main reasons for work leave. As suggested by Bernardes and Cole [22,23], macroargonomic methods should be used in ergonomic studies to reduce musculoskeletal disorders.

The HTA results showed that the Soldering System takes the most time of the workers (more than three hours per day), then working with hand tools was the second time consuming task and the third time consuming task was wind tools which take about one hours of workers.

In the proposed designs of ergonomic workstations of assemblers, numerous studies have shown that the most effective way to prevent occupational injuries is to minimize risks of injuries through modification of workstation design.

Based on evaluations in different assembly halls, the assembly process divided into two main tasks 1) light assembly including work with light electronic boards weighted less than two kilograms which was run by women, and 2) heavy assembly – matters related to closing boards on main chassis weighted more than forty kilograms which was held by men. The proposed ergonomic design of the assembler workstation was done separately for these two parts.

**Figure 4:**
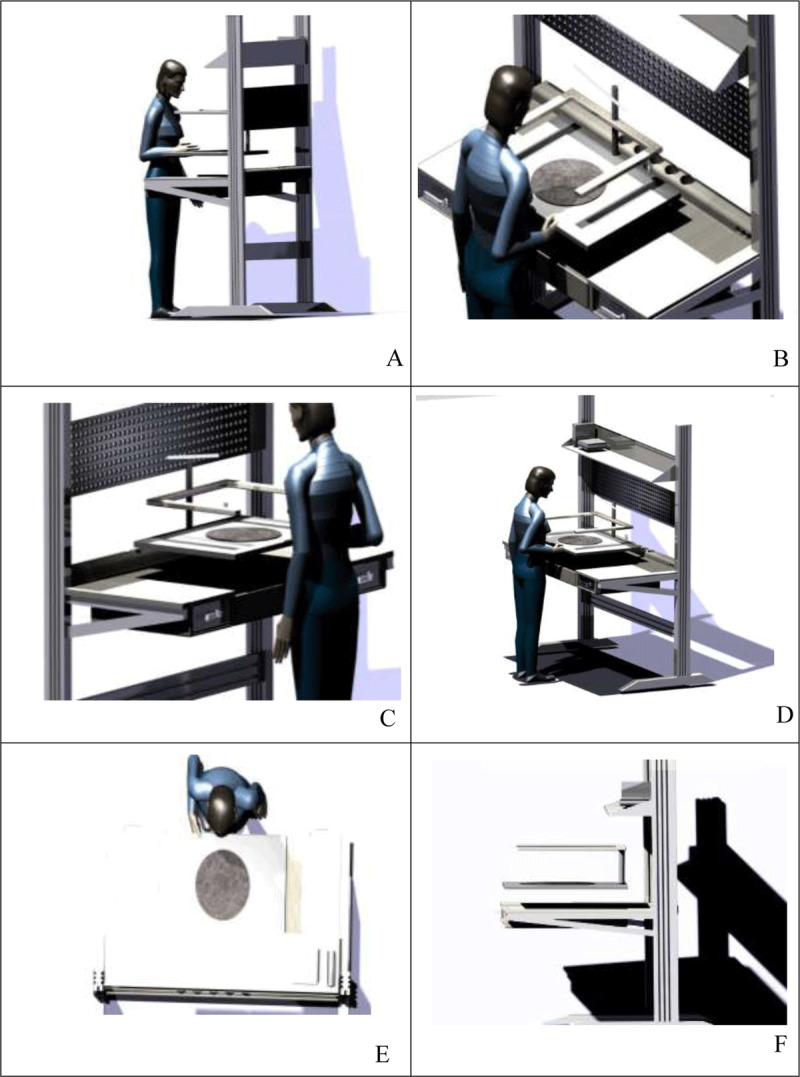
Different views of the proposed design for the lightweight assembly plant.

Light Assembly: As it is readily apparent in ***Figure 4***, holes are designed at the end of the table surface that can be a place to store tools in different sizes, even hot saucers, drink glasses and small pots. The small holes at the bottom are needed for power outlets for LED lamps, lighters, electron microscopes (***Figure 4***).

Heavy assembly: This part is applied only for men in the assembly hall. Therefore, based on the required anthropometric dimensions of individuals accompanied with the dimensions of the assembly parts, the length of the table was increased to 180 cm.

**Figure 5:**
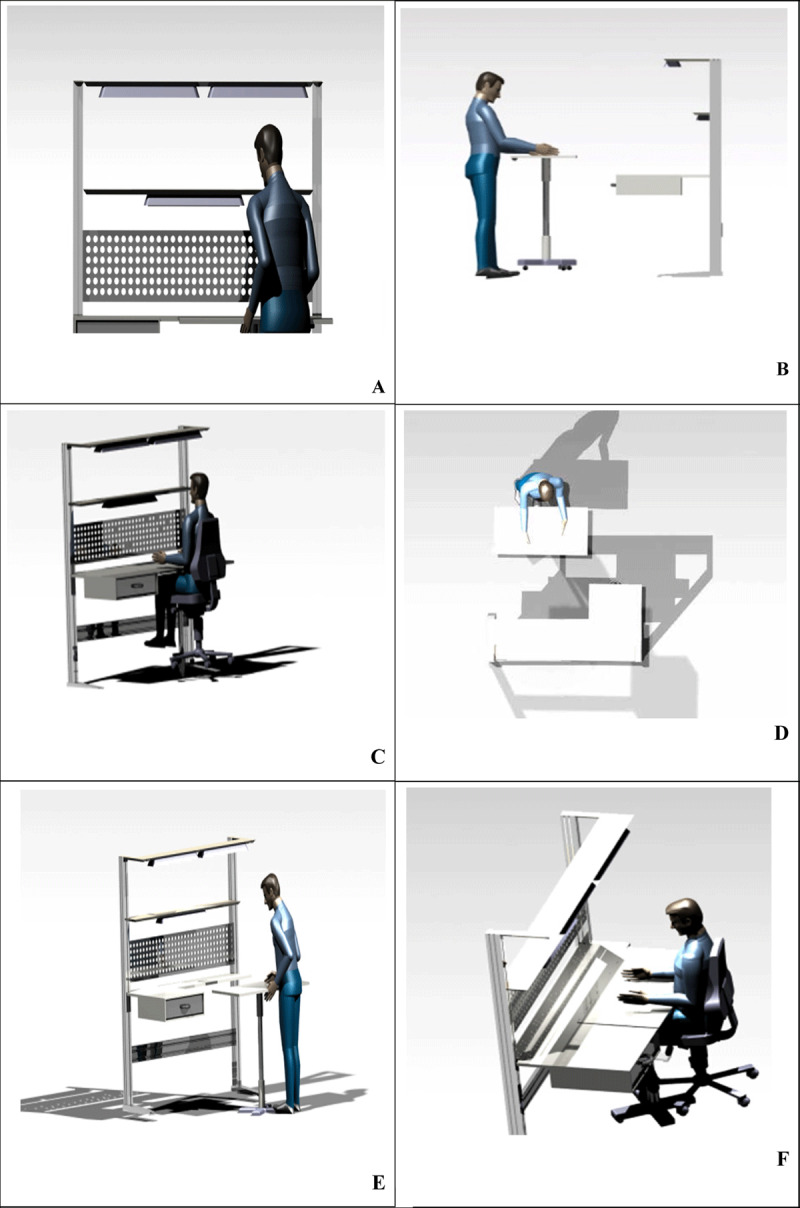
Different views of the proposed design for the heavy assembly workstation.

In this workstation, as it has been mentioned in the previous section, an aluminum perforated surface is installed in the middle of the table bases where a person can install tools or put stationery and even his daily notes on. Of course, the metal surface in this section is thicker and has large holes where workers can hold their heavier electronics and wind instruments (***Figure 5***).

In this study, an attempt was made to design a standing-sitting workstation to reduce static work fatigue and the risk of musculoskeletal disorders in assemblers. It has been more dynamic and changeable.

Inconsistent with the findings of the study carried out by Dickhout [24], those workstations that have different muscle demands, such as workers who have job rotation, have less job fatigue. Also, the finding of Cui showed that standing posture uses less muscle than sitting posture, although the neck-shoulder muscle responds differently perform in a sitting or standing position [25].

Therefore, the designs were made to reduce musculoskeletal disorders, the posture of work from fully sitting change to standing-sitting, and finally, a dynamic workstation to use different muscles of the body during work.

Findings from field studies showed that there are many physical, environmental, and ergonomic factors in the work environment, each of which can have a significant impact on the health and performance of assemblers. The findings of the present study led to the design of an ergonomic and dynamic workstation for assemblers.

The results of the evaluations showed that in the design of most workstations of assemblers in Shiraz electronics industries, ergonomic principles were not completely considered and the implementation of targeted ergonomic interventions is necessary. The proposed designs can be used as a template for the design and redesign of the assembly plant workstation.
